# Studying complexity in health services research: desperately seeking an overdue paradigm shift

**DOI:** 10.1186/s12916-018-1089-4

**Published:** 2018-06-20

**Authors:** Trisha Greenhalgh, Chrysanthi Papoutsi

**Affiliations:** 1 Nuffield Department of Primary Care Health Sciences, University of Oxford, Oxford, UK; 2 Radcliffe Primary Care Building, Radcliffe Observatory Quarter, Woodstock Road, Oxford, OX2 6GG UK

**Keywords:** Complexity, Systems thinking, Methodology, Healthcare

## Abstract

Complexity is much talked about but sub-optimally studied in health services research. Although the significance of the complex system as an analytic lens is increasingly recognised, many researchers are still using methods that assume a closed system in which predictive studies in general, and controlled experiments in particular, are possible and preferred. We argue that in open systems characterised by dynamically changing inter-relationships and tensions, conventional research designs predicated on linearity and predictability must be augmented by the study of how we can best deal with uncertainty, unpredictability and emergent causality. Accordingly, the study of complexity in health services and systems requires new standards of research quality, namely (for example) rich theorising, generative learning, and pragmatic adaptation to changing contexts. This framing of complexity-informed health services research provides a backdrop for a new collection of empirical studies. Each of the initial five papers in this collection illustrates, in different ways, the value of theoretically grounded, methodologically pluralistic, flexible and adaptive study designs. We propose an agenda for future research and invite researchers to contribute to this on-going series.

## Introduction

Medicine’s interest in complexity has, to date, been largely superficial, both theoretically and empirically. It is fashionable to talk of complex interventions, complex systems, complex patients, wicked problems, and the like. However, with few exceptions, we embrace the theme of complexity in name only and fail to engage with its underlying logic.

In 2001, Plsek and Greenhalgh edited a series of articles in the *British Medical Journal*, introducing the topic of complexity [1] and applied complexity principles to clinical care [2], leadership and management [3] and lifelong learning [4]. That series was extensively cited, yet the paradigm shift it heralded did not happen.

Contemporary healthcare is experiencing several important challenges, including a mismatch between the ‘patient in the guideline’ and the ‘patient in the bed’ due to multi-morbidity and interacting sociocultural influences; an inability for ‘marginalised’ patients to access GP services despite the super-science miracle cures ubiquitous in the media; new staff roles, organisational forms and technologies that sometimes seem to worsen the very problems they were introduced to solve; and the policy sacred cow of integrated care repeatedly proving impossible to deliver in practice. As these challenges become ever more pressing, it is time to revisit the original question asked by the 2001 *British Medical Journal* series: “What is complexity and what are its implications for clinical practice, research, service organisation and professional education?”

Today, we launch a new series inspired by an international workshop “We Need to Talk about Complexity” held in Oxford, UK, in June 2017 [5], which prompted an open call for papers by *BMC Medicine* [6]. The series begins with five papers [7–11], and more will be added in the future. We hope that this new series will (1) explain what complexity thinking is and how it challenges some of the deeply-held assumptions held by the medical community about how the world works; (2) illustrate how complexity-informed research and scholarship can provide insights and ways forward for some of medicine’s most intractable problems; and (3) outline a future research agenda for the study of complexity in medicine and healthcare.

## Complexity and complex systems

Complexity is described as “*a dynamic and constantly emerging set of processes and objects that not only interact with each other, but come to be defined by those interactions*” [12]. Complex systems have fuzzy boundaries; their interacting agents operate on the basis of internal rules that cannot always be predicted; and they adapt, interact and co-evolve with other systems [1, 13, 14]. Crucially, complexity is a feature of the system(s), not merely a characteristic of interventions [15, 16]. Indeed, whether an intervention is simple (one active component, unchanging) or complex (multiple interacting components), the ‘system’ in which the intervention is implemented will almost invariably need to adapt in some way to accommodate it [15, 16]. Often, a planned intervention (e.g. a multi-component public health programme that aims to prevent type 2 diabetes) and its context (e.g. a deprived, multi-ethnic inner city community with limited leisure facilities, multiple fast-food and street-food outlets, and a variety of existing faith-based community support programmes) will be inter-related and reciprocally interacting – the dancer and the dance are intertwined.

Other, less widely discussed, features of complexity are highly relevant to the study of health services and systems. The world moves quickly; baselines shift; technologies crash; actions are (variously) constrained; and certainty is elusive. The gap between the evidence-based ideal and the political and material realities of the here-and-now may be wide. Decisions must be made on the basis of incomplete or contested data. People use their creativity and generate adaptive solutions that make sense locally. The articulations, workarounds and muddling-through that keep the show on the road are not footnotes in the story, but its central plot. They should be carefully studied and represented in all their richness.

These core characteristics of complex systems suggest that the randomised controlled trial (in which the effects of context are ‘controlled for’) will address only a fraction of the unanswered questions relating to healthcare organisations and systems [12, 13, 17]. Because the system is dynamic (turbulent, even), the conventional scientific quest for certainty, predictability and linear causality must be augmented by the study of how we can best deal with uncertainty, unpredictability and generative causality. For this, we need research designs and methods that foreground dynamic interactions and emergence – most notably, in-depth, mixed-method case studies that can act as concrete, context-dependent exemplars, including powerful ethnographic narratives paying attention to interconnectedness and incorporating an understanding of how systems come together as a whole from different perspectives [12, 18].

The original Medical Research Council (MRC) framework for the development and testing of complex interventions, published in 2001, defined these as consisting of multiple components acting independently and inter-dependently, in such a way that made it difficult to identify the ‘active ingredient’ [19]. This early guidance proposed a structured approach to developing such interventions and upheld the randomised controlled trial as the gold standard for testing them. An update to the MRC framework in 2008 [20] extended the definition of complexity to include the degree of behavioural change and level of organisational involvement required to implement the intervention, the level of variability of outcomes and the degree of intervention flexibility needed. Another update in 2015 [21] highlighted the importance of non-linearity and iterative local tailoring, and placed substantially more emphasis on the need for non-experimental, mixed methods and process-based approaches for studying such phenomena.

The MRC’s approach to complexity has thus shifted considerably – both in terms of where the complexity is assumed to lie (from the intervention to the system to the interaction between the two) and in relation to how best to study it (from the randomised controlled trial atop an assumed hierarchy of evidence to a genuinely pluralistic approach that gives appropriate weight to real-world case studies). The MRC’s latest position introduces (although does not consistently uphold) an underlying philosophical shift from a conventional Newtonian (linear, cause-effect) perspective to a systems perspective that embraces non-linear causality (Table 1). However, many research funders, principal investigators and journal editors remain wedded to the intervention-focused approach to complexity as originally mooted by the MRC. Unfortunately, ‘complexity research’ has come to be equated in some circles with a highly standardised sequence of developing a structured, multi-component intervention, testing it in a randomised controlled trial and following a somewhat formulaic and prescriptive approach to implementation [13, 22–24].

**Table 1:** Traditional versus new paradigm approaches to researching health services and systems

	Traditional approach	New paradigm (complexity-informed) approach
Goal of research	Establishing the truth, universal and enduring; finding solutions to well-defined problems	Exploring tensions; generating insights and wisdom; exposing multiple perspectives; viewing complex systems as moving targets
Assumed model of causality	Linear, cause-and-effect causality (perhaps incorporating mediators and moderators)	Emergent causality: multiple interacting influences account for a particular outcome but none can be said to have a fixed ‘effect size’
Typical format of research question	“What is the effect size of the intervention on the predefined outcome, and is it statistically significant?”	“What combination of influences has generated this phenomenon? What does the intervention of interest contribute? What happens to the system and its actors if we intervene in a particular way? What are the unintended consequences elsewhere in the system?”
Mode of representation	Attempt to represent research in one authoritative voice	Attempt to illustrate the plurality of voices inherent in the research and phenomena under study
Good research is characterised by	Methodological ‘rigour’, i.e. strict application of structured and standardised design, conventional approaches to generalisability and validity	Strong theory, flexible methods, pragmatic adaptation to emerging circumstances, contribution to generative learning and theoretical transferability
Purpose of theorising	Disjunctive: simplification and abstraction; breaking problems down into analysable parts	Conjunctive: drawing parts of the problem together to produce a rich, nuanced picture of what is going on and why
Approach to data	Research should continue until data collection is complete	Data will never be complete or perfect; decisions often need to be made in situations of incomplete or contested data
Analytic focus	Dualisms: A versus B; influence of X on Y	Dualities: inter-relationships and dynamic tensions between A, B, C and other emergent aspects

It is time we faced the irony of this situation. There are no universal solutions to the challenges of complex health systems, nor is there a set of universal methods that will bring us closer to the truth. Research protocols consisting entirely of pre-ordained work packages arranged around a boxes-and-arrows diagram accompanied by tight milestones and timelines may be the stuff that funding panels’ dreams are made of, but when the focus of inquiry is the health system, such approaches are – almost by definition – less likely to generate meaningful findings than studies which engage pragmatically with the multiple uncertainties involved and offer a flexible and emergent approach to exploring them.

## Understanding complexity in health systems: International perspectives

The initial five articles in this series report on studies of healthcare delivery and health systems. The empirical topics are diverse; they cover mental health services, respiratory conditions, medicines management, hospital-based rapid response teams, system-level accreditation mechanisms and digital health solutions (such as video consultations, assisted living technologies and remote monitoring).

In the first paper, Braithwaite et al. [7] challenge traditional thinking on implementation science as based, to a greater or lesser extent, on linear and mechanistic (‘pipeline’) models of knowledge translation. Drawing on systems thinking in social and organisational science, the authors discuss how implementation can be understood as an emergent and dynamic process. To achieve system-level change, complexity-informed approaches to implementation would need to depart from a narrow focus on intervention fidelity to embrace effective adaptation and tailoring to context, working closely with local stakeholders, and viewing implementation as an iterative, recursive and long-term process. Taking the example of two large-scale system transformations in Australia (implementation of rapid response systems and introduction of quality standards for health services accreditation), the paper argues that quality and safety improvement can be achieved by ‘attending to’, rather than trying to ‘control for’, complexity.

Wolpert and Rutter [8] address what is often seen as the cornerstone of evaluation and improvement – routinely collected quantitative datasets. Their paper raises important questions about the value and usefulness of such datasets with regards to measuring and representing change in complex health systems. As the authors argue, quantitative datasets invariably contain Flawed, Uncertain, Proximate and Sparse (FUPS) data, which either become over-interpreted (leading to unwarranted conclusions) or end up being dismissed as incomplete and unreliable. The authors propose a third option, namely to embrace FUPS datasets – warts and all – as a key contributor in the change effort, recognising that, whilst they cannot fully capture the complex world they represent, they still have the potential to expose issues for interrogation, act as sensitising devices for developing understanding and mobilise uncomfortable knowledge. The paper includes an important list of principles for analysing and facilitating discussion on the basis of FUPS data, illustrating these through an empirical example in UK child mental healthcare.

Greenhalgh et al.’s [10] article addresses complexity as manifested in technological innovation. Drawing on an extensive empirical dataset from six contrasting case studies of technology-supported change in health and social care, they present an evidence-based, theory-informed, and pragmatic framework to explain the Non-adoption or Abandonment of technology by individuals and difficulties achieving Scale-up, Spread and Sustainability (NASSS) in organisations [25]. The NASSS framework embraces multiple levels of analysis to help predict and evaluate programme success. Technology failures, partial successes and unanticipated problems are explained by teasing out the multiple aspects of complexity across interacting domains, including the condition, technology, value proposition, adopter system, organisation, wider context and temporal change. The discussion section includes recommendations for both reducing the complexity of a technology-supported change programme and ‘running with’ aspects of complexity that cannot be reduced.

Long et al. [9] offer a perspective of how to engage with complexity in practice, based on synergies between complexity theory and pragmatist philosophy. These authors depict pragmatism as a way of prioritising actionable knowledge linked to its contexts of use and suited to address practical questions. Their analysis draws on a 3-year project aimed at developing simulation models to provide strategic decision support for senior leaders in a large public mental health service in Australia. Through a study of simulation modelling to support service implementation and evaluation, they illustrate how complexity theory and pragmatism can be used as complementary approaches to guide and iteratively enhance understanding. Their analysis focuses primarily on the role of agency (that is, human initiative and action), emergent outcomes (things that happen as a result of multiple unfolding events and phenomena), continuous learning and adaptive use of methods. This paper also questions prevailing assumptions about complexity and suggests there is a need to further explore its philosophical and epistemological underpinnings.

Reed et al. [11] respond to the call for complexity-informed approaches in healthcare by synthesising learning from an extensive programme of improvement initiatives in the UK. Their contribution introduces the empirically-driven and theory-supported framework on Successful Healthcare Improvement From Translating Evidence (SHIFT-Evidence). Through analytical auto-ethnography and grounded theory analysis on data collected across 22 quality improvement projects over a 5-year period, the authors foreground the emergent behaviour of complex systems and the iterative, adaptive nature of change. Their analysis discusses tensions in embedding evidence-based practices against local constraints (‘acting scientifically and pragmatically’), the importance of recognising and appreciating the complexity of systemic issues (‘embracing complexity’), and the need to facilitate commitment and engagement from important stakeholders (‘engaging and empowering’). These three principles are illustrated in two examples of improvement projects in community-acquired pneumonia and medicines management. Grounded in the practical reality of healthcare improvement, the SHIFT-Evidence framework is also accompanied by 12 ‘simple rules’ to guide evidence translation.

The articles included in this collection illustrate the value of iterative research approaches that are theoretically grounded, methodologically pluralistic, flexible and ecologically focused. They adopt a range of approaches to produce grounded explanations of what happened when someone attempted to achieve change in a complex, fast-changing healthcare environment. None of the papers offers simple solutions, predictive tools or universal formulae (though some submissions that were rejected from this call did purport to ‘solve’ complexity in this way). This series builds on the creative work of other research teams who have taken a nuanced and theory-informed approach to the study of complexity in healthcare - for example, Dixon-Woods et al. [26] on ex-post theorising of patient safety initiatives, Nugus et al. [27] on integrated care in the emergency department, and Lanham et al. [28] on scale-up and spread in healthcare.

All these studies engage, in different ways, with what Tsoukas has called ‘conjunctive theorising’, that is, avoiding the temptation to simplify and abstract (an approach which Tsoukas calls ‘disjunctive theorising’), towards an approach to theory that generates rich pictures of complex phenomena by drawing together different kinds of data from multiple sources [29]. Conjunctive theorising, proposes Tsoukas, is characterised by an open-world ontology (viewing the world as subject to multiple interacting influences, and recognising that it serves no useful analytic purpose to strip away these layers of influence in artificial simplifications), a performative epistemology (that is, a focus on real-world action and on what becomes possible through action), and a poetic praxeology (that is, a way of writing up case studies that values descriptive detail, apt metaphor and narrative coherence).

The case studies in this series suggest a number of high-level themes that could be explored further in future research calls. First, new research could address the general proposal that the health services research community should embrace a richer and more diverse methodological repertoire when researching complex systems. How, specifically, might that methodological repertoire be extended? Second, new research could respond to Tsoukas’ call for a retreat from simplification and abstraction, and explore how conjunctive theorising could extend the possibilities of the mixed-method case study [29]. Third, research in real-world settings could generate new ways of working productively with imperfect (FUPS) data [8]. Fourth, as Long et al. [9] have shown, there is much potential still to be explored in relation to the use of simulation modelling in the study of complexity in healthcare. Finally, we need to put more effort into developing theory-driven and empirically focused frameworks that can guide implementation and evaluation from a complexity perspective [10, 11].

## Conclusion

Law and Mol were right to suggest that “*We need other ways of relating to complexity, other ways for complexity to be accepted, produced, or performed*” [30]. As researchers, clinicians and lifelong learners, we need to develop capability and capacity to handle the unknown, the uncertain, the unpredictable and the emergent [4]. In other words, we need to develop a ‘systems mindset’ that recognises changing interrelationships between parts of the system (or even what constitutes a system at any given time) and adapts to unexpected change [31]. Complexity science will not provide a simple fix for the inherent tensions and paradoxes in contemporary health systems, but it will allow us to focus on – and begin to research – uncomfortable knowledge, to negotiate good compromises and to embrace creative, reflexive and collaborative ways of working and thinking. The organising vision behind complexity-informed healthcare research needs to encompass “*a commitment to engage in disagreements*” [32], making sure that we remain critical about our assumptions and methods. We invite readers to share this commitment by continuing to contribute to this thematic collection. Submissions are open until June 30, 2019.
